# Breastfeeding success and perceived social support in lactating women with a history of COVID 19 infection: a prospective cohort study

**DOI:** 10.1186/s13006-023-00601-0

**Published:** 2023-12-04

**Authors:** Ashraf Moini, Fatemeh Heidari, Mitra Eftekhariyazdi, Reihaneh Pirjani, Marjan Ghaemi, Nasim Eshraghi, Maryam Rabiei

**Affiliations:** 1 Department of Obstetrics and Gynecology, Endocrinology and Female Infertility Unit, Arash Women’s Hospital, Tehran University of Medical Sciences, Tehran, Iran; 2 Breast Disease Research Center (BDRC), Tehran University of Medical Sciences, Tehran, Iran; 3 Department of Endocrinology and Female Infertility, Reproductive Biomedicine Research Center, Royan Institute for Reproductive Biomedicine, ACECR, Tehran, Iran; 4 Department of Obstetrics and Gynecology, School of Medicine, Sabzevar University of Medical Sciences, Sabzevar, Iran; 5 Vali-E-Asr Reproductive Health Research Center, Family Health Research Institute, Tehran University of Medical Sciences, Tehran, Iran

**Keywords:** COVID 19, Pregnancy, Breastfeeding

## Abstract

**Background:**

Given the limited availability of research on the association between COVID-19 infection and breastfeeding success, the primary objective of this study is to conduct a comprehensive evaluation of this relationship.

**Methods:**

This prospective cohort study included 260 women who were on the postnatal ward of an academic hospital affiliated with Tehran University of Medical Sciences during the COVID-19 pandemic (between March and August 2021). Among these women, 130 had tested positive for COVID-19 in pregnancy, while the remaining 130 were considered healthy. The study aimed to assess various factors, including sociodemographic characteristics and the results of four validated questionnaires: The Bristol Breastfeeding Questionnaire, The Multidimensional of Perceived Social Support (MPSS), The Breastfeeding Self-Efficacy Scale (BSES), and The Postpartum Partner Support Scale (PPSS). These questionnaires were administered to each participant to gather relevant data. After eight weeks, a telephone follow-up was carried out to assess the success of breastfeeding. The evaluation focused on determining if exclusive breastfeeding was maintained or not. Data was collected by questioning mothers about their infants’ feeding habits in the past 24 h. Exclusive breastfeeding refers to the exclusive use of breast milk without the introduction of other liquids or solid foods.

**Results:**

Women with a previous COVID-19 infection (case group) had a lower mean infant gestational age (*P* < 0.001) and a higher prevalence of cesarean section (*P* = 0.001) compared to the control group. The proportion of women who exclusively breastfed was higher in the control group (98.5%) than in women with a history of COVID-19 infection (89.2%) (*P* = 0.011). Furthermore, the case group reported lower scores in perceived social support and the Breastfeeding Self-Efficacy Scale, in contrast to the control group. Notably, there was a significant correlation between breastfeeding success and women’s breastfeeding self-efficacy score.

**Conclusions:**

The findings of this study offer valuable insights for healthcare professionals, enabling them to promote early initiation of breastfeeding in mothers with a history of COVID-19 infection, while ensuring necessary precautions are taken.

## Background

Breastfeeding rates during the COVID-19 lockdown have been the subject of several studies comparing them to the pre-pandemic period [1]. According to a retrospective study conducted by Koleilat in Southern California, the prevalence of any breastfeeding at six months significantly decreased following March 2020, with rates dropping from 49 to 39% [2]. A Canadian study emphasized that lactating women faced challenges due to inadequate care provided during their hospital stay, the absence of social support, and their own poor mental health [3]. Consequently, some of these mothers had to discontinue breastfeeding earlier than they had intended, going against their desired duration [3].

To date, research on the association between COVID-19 infection and breastfeeding success remains limited. Recent studies have sought to investigate the impact of COVID-19 infection on various aspects of breastfeeding, such as initiation, duration, and exclusivity. A systematic review study suggested a potential predominantly negative influence of COVID-19 infection on breastfeeding success, although there were a few instances where certain mothers viewed the lockdown positively as it provided protection for the bond between mother and infant [4]. Understanding the potential risks and benefits of breastfeeding during the pandemic is crucial for healthcare providers and mothers to make informed decisions regarding infant feeding practices.

One of the primary concerns during the COVID-19 pandemic is the possibility of transmitting the virus through breast milk [5]. Initially, there were reports of detecting SARS-CoV-2 viral RNA in breast milk samples of infected mothers, which led to concerns regarding potential transmission via breastfeeding [6]. However, subsequent studies indicate that the risk of transmission through breast milk is minimal [7, 8].

The stress and anxiety brought about by the pandemic, along with the disruptions to healthcare services, have the potential to adversely affect breastfeeding outcomes [9]. A published review revealed that mothers who tested positive for COVID-19 were less inclined to initiate breastfeeding and had a shorter duration of exclusive breastfeeding in comparison to mothers who tested negative [4].

Furthermore, the utilization of personal protective equipment while breastfeeding can present difficulties for both mothers and healthcare providers, potentially affecting the overall breastfeeding experience. Concerns about transmitting the virus to their infants may also lead some mothers to feel hesitant about breastfeeding, while others may face separation from their infants due to hospital policies related to COVID-19 [10].

Understanding the potential risks and benefits of breastfeeding during the pandemic is crucial for healthcare providers and mothers to make informed decisions about infant feeding practices. The objective of this study was to investigate the association between COVID-19 infection and breastfeeding success.

## Method

### Study setting

This prospective cohort study was conducted between March and August 2021, during the COVID-19 pandemic. It involved 260 women recruited on the postnatal ward of an academic center affiliated with Tehran University of Medical Sciences. Among them, 130 women had tested positive for SARS-CoV-2 in pregnancy based on a positive nasopharyngeal swab, and gave birth at 34 to 41 weeks. Additionally, 130 healthy women were randomly selected to serve as the control group.

### Eligibility criteria

The inclusion criteria for this study encompassed breastfeeding women aged between 20 and 40 years-old who received breastfeeding education, tested positive for COVID-19 but did not require ICU admission, had no history of severe postpartum depression or other psychological problems. Exclusions from the study involved women with preterm neonates requiring neonatal intensive care unit (NICU) admission, contraindications to breastfeeding, previous unsuccessful breastfeeding attempts, underlying maternal disorders that could impact breastfeeding, and the current use of illicit drugs.

### Ethical consideration

This study was approved by the ethics committee of Tehran University of medical sciences (IR.TUMS.MEDICINE.REC.1400.530). Eligible women provided written informed consent before they were enrolled in this study. The participants’ information was collected securely and solely used for the purpose of this study.

### Data measures

Sociodemographic and obstetrics information including maternal age, gravidity, parity, baby’s gestational age (based on week), mode of delivery and the number of live children were collected. For this study, we utilized four validated questionnaires included The Bristol Breastfeeding questionnaire, The Multidimensional of Perceived Social Support (MPSS), The Breastfeeding Self-Efficacy Scale (BSES) and The Postpartum Partner Support Scale (PPSS). In addition, telephone follow-up was conducted eight weeks later to evaluate the success of breastfeeding, assessing whether it remained exclusive or not. In this study, our objective was to assess exclusive breastfeeding based on the World Health Organization (WHO) recommendation [11, 12]. We collected data by asking mothers about their infant’s feeding practices within the previous 24 h. Exclusive breastfeeding is defined as the practice of feeding an infant solely with breast milk, without introducing any other liquids or solid foods [13–15].

The PPSS designed by Dennis et al. consists of 20 items that were rated on a 4-point Likert scale, ranging from “strongly disagree” to “strongly agree” [16]. This inventory assessed general partner support and the Iranian version of the PPSS questionnaire has been found to demonstrate good internal consistency and reliability, as confirmed by Eslahi et al. [17].

The MPSS is a 12-item checklist, rated on a 7-point Likert scale, that evaluates perceived social support from friends, family and significant other [18]. Salimi et al. demonstrated that MSPSS is a valid and reliable assessment tool for Iranian population [19].

The Bristol Breastfeeding questionnaire, developed by Ingram et al., is an assessment tool for evaluating different aspects of efficient breastfeeding, including infant positioning, attachment, sucking, swallowing, and comfort. The Cronbach’s alpha coefficient for the Bristol Breastfeeding Scale was reported as 0.96, indicating high internal consistency [20].

The Breastfeeding Self-Efficacy Scale (BSES) is a checklist consisting of 14 items that measure maternal confidence in her ability to breastfeed her infant, using a 5-point Likert-type scale [21]. The validity and reliability of this questionnaire among Iranian women were confirmed by Araban et al. [10, 22].

### Data analysis

Data analysis was performed using SPSS software (version 26, SPSS, Chicago, IL, USA). A comparison of the total scores for each questionnaire was conducted between two groups: lactating women with a history of COVID-19 infection and healthy lactating women. For this comparison, an independent t-test or Mann-Whitney U test was employed, as appropriate. The Pearson Correlation Coefficient was used to assess the relationship between breastfeeding wellbeing and questionnaire scores. Maternal factors were compared between the two groups using either a chi-square test or Fisher’s exact test. The significance level was set at *P* < 0.05.

## Results

A total of 260 women participated in the study, including 130 lactating women with a history of COVID-19 infection and 130 healthy women as a control group. Table 1 presents the maternal and obstetrical information of the participants. There were no significant differences observed in maternal age and gravidity between the two groups. However, the mean infant’s gestational age was significantly lower (*P* < 0.001) in the case group, and the rate of cesarean section was significantly higher (*P* = 0.001) compared to the control group.

**Table 1:** Maternal and obstetrics characteristics of the women with a history of COVID 19 infection (case group) compared to healthy women (control group)

Characteristics		Case (n = 130)	Control (n = 130)	*P* value
Maternal age (year) (mean ± SD)		29.4 ± 5.1	29.19 ± 5.2	0.6^1^
Gestational age in delivery (week) (mean ± SD)		37.8 ± 1.4	38.7 ± 1.9	< 0.001^1^
Cesarean section rate (n (%))		60 (46.1)	47 (36.1)	0.001^2^
Gravidity (n (%))	1	24 (18.4)	28 (21.5)	0.1^2^
	2	71 (54.6)	58 (44.6)	
	3	35 (26.9)	44 (33.8)	
Breastfeeding status (n (%))	Exclusive	116 (89.2)	128 (98.4)	0.011^3^
	Non exclusive	14 (10.7)	2 (1.5)	

The t-test was utilized^1^. The Pearson chi-square test was used^2^. The Fisher’s exact test was used^3^

Regarding breastfeeding practices, there was a notable difference observed between the two groups. The percentage of women practicing exclusive breastfeeding was significantly higher among the healthy women (98.46%) compared to the lactating women with a history of COVID-19 infection (89.23%) (*P* = 0.011).

The mean score of the Bristol Breastfeeding scale was not significantly different between the two groups, suggesting that the success of breastfeeding among mothers in the study group was comparable to that in the control group. Additionally, the PPSS score showed no significant difference in the case group. However, the results revealed that the MPSS score and BESE score were significantly higher in the control group compared to the case group (*P* = 0.001) (Table 2).

**Table 2:** Total scores for questionnaires for women with a history of COVID 19 infection (case group) compared to healthy women (control group)

Questionnaires	Case (n = 130) (mean ± SD)	Control (n = 130) (mean ± SD)	*P* value
The Multidimensional of Perceived Social Support score	63.9 ± 11.2	69.2 ± 12.4	0.001
The Postpartum Partner Support Scale score	63.9 ± 6.2	65.2 ± 7.2	0.1
The Breastfeeding Self-Efficacy Scale score	51.0 ± 10.4	55.0 ± 9.7	0.001
The Bristol Breastfeeding scale score	6.0 ± 2.12	6.0 ± 2.12	0.7

T-test was utilized to evaluate the relationship between numerical variables

The results of the Pearson correlation coefficient analysis among women who had a history of COVID-19 disease in late pregnancy indicated that there was no significant correlation between the success of breastfeeding and maternal characteristics, MPSS score, and PPSS score. However, a significant positive correlation was observed between successful breastfeeding and breastfeeding self-efficacy (r = 0.5, *P* = 0.001) (as shown in Table 3).

**Table 3:** Pearson correlation coefficient analysis in women who had history of COVID 19

Characteristics	r	*P* value
Maternal age (year)	0.05	0.5
Infant’s gestational age (week)	0/08	0.4
Cesarean section	0.08	0.2
Successfully breastfeeding	0.3	0.001
MPSS score	− 0.03	0.6
PPSS score	0.1	0.2
BSES score	0.5	0.032

## Discussion

We found a significant difference in the proportion of women practicing exclusive breastfeeding between the control group and the group of women infected with COVID-19 during late pregnancy. The control group showed a higher proportion of women engaging in exclusive breastfeeding compared to the infected group. Additionally, the study revealed that the infected group had lower scores in perceived social support and Breastfeeding Self-Efficacy Scale compared to the control group. Notably, among women with a history of COVID-19 infection, there was a significant correlation between the success of breastfeeding and their breastfeeding self-efficacy scores. This suggests that higher levels of self-efficacy in breastfeeding were associated with a greater likelihood of successful breastfeeding in women who had contracted COVID-19 during late pregnancy.

In light of the COVID-19 pandemic, the World Health Organization (WHO) recommends that breastfeeding should be initiated or continued by women with COVID-19 infection, with necessary precautions such as wearing a mask and practicing proper hand hygiene before and after breastfeeding [11].

Previous studies have shown that the transmission of COVID-19 disease through breastmilk is rare or non-existent [10, 23]. However, our research reveals a notable difference in the rate of exclusive breastfeeding between lactating women with a history of COVID-19 infection and healthy lactating women. Our findings align with other studies that have also reported lower rates of exclusive breastfeeding among mothers affected by COVID-19 disease [24, 25].

The lower rate of exclusive breastfeeding among mothers with COVID 19 infection can be attributed to various factors. One significant factor is the increased postpartum maternal anxiety levels due to the stress and uncertainty surrounding the infection. Healthcare providers also contribute to this issue as they express concerns about the potential contamination of breastmilk in women who have had COVID-19 [26]. During the COVID-19 outbreak, pregnant and breastfeeding women experienced heightened levels of stress and anxiety. These emotional challenges have been shown to have a negative impact on both maternal and neonatal outcomes [27, 28]. Previous studies have highlighted the importance of maternal mental health and well-being as predictors of breastfeeding success [29–31].

Additionally, this study revealed that lactating women who had previously experienced a COVID-19 infection reported significantly lower levels of perceived social support and breastfeeding self-efficacy when compared to healthy lactating women. This finding aligns with the results of a cross-sectional study conducted by Piankusol et al., which demonstrated that reduced family support during the COVID-19 lockdown was associated with a decrease in exclusive breastfeeding rates among lactating women [32].

A recent review conducted by Pacheco et al. highlighted the negative effects of separating mothers from their infants to prevent the transmission of COVID-19. This separation has been found to have detrimental effects on the initiation and duration of breastfeeding, ultimately impacting maternal mental health and overall well-being [33].

Therefore, it is crucial for healthcare providers to consistently support and encourage breastfeeding among mothers who have contracted COVID-19. However, it is equally important for these providers to implement appropriate precautions to minimize the risk of transmitting the virus.

This study has limitations that should be acknowledged. Firstly, the number of participants was small, which may restrict the generalizability of the findings. Secondly, in the case group, the rate of cesarean section was significantly higher, and infected women had a notably lower mean infant’s gestational age compared to the control group. These factors have the potential to influence the rate of exclusive breastfeeding. However, despite these limitations, we employed various validated questionnaires to assess breastfeeding success and maternal mental health. This approach enabled us to gather valuable information that can be utilized by healthcare providers in assisting pregnant women to achieve successful exclusive breastfeeding.

## Conclusions

Lactating women with a history of COVID-19 infection, who sought care at our academic center, exhibited lower rates of exclusive breastfeeding, as well as lower levels of perceived social support and breastfeeding self-efficacy, when compared to healthy lactating women. These findings emphasize the importance of healthcare providers offering tailored support and counseling to mothers who have experienced COVID-19 infection, in order to facilitate positive breastfeeding outcomes. It is crucial to conduct further research to investigate the effects of COVID-19 on breastfeeding during the first six months of an infant’s life, including the potential long-term consequences associated with reduced rates of exclusive breastfeeding.

## Data Availability

Data is available upon request.

## References

[1] Turner S, McGann B, Brockway MM (2022). A review of the disruption of breastfeeding supports in response to the covid-19 pandemic in five western countries and applications for clinical practice. Int Breastfeed J.

[2] Koleilat M, Whaley SE, Clapp C (2022). The impact of covid-19 on breastfeeding rates in a low-income population. Breastfeed Med.

[3] Rice K, Williams S (2021). Women’s postpartum experiences in Canada during the covid-19 pandemic: a qualitative study. Can Med Association Open.

[4] Lubbe W, Niela-Vilén H, Thomson G, Botha E (2022). Impact of the covid-19 pandemic on breastfeeding support services and women’s experiences of breastfeeding: a review. Int J Women’s Health.

[5] Groß R, Conzelmann C, Müller JA, Stenger S, Steinhart K, Kirchhoff F (2020). Detection of sars-cov-2 in human breastmilk. Lancet.

[6] He YF, Liu JQ, Hu XD, Li HM, Wu N, Wang J (2023). Breastfeeding vs. breast milk transmission during covid-19 pandemic, which is more important?. Front Pead.

[7] Chambers C, Krogstad P, Bertrand K, Contreras D, Tobin NH, Bode L (2020). Evaluation for sars-cov-2 in breast milk from 18 infected women. JAMA.

[8] Pérez-Bermejo M, Peris-Ochando B, Murillo-Llorente MT. Covid-19: relationship and impact on breastfeeding-a systematic review. Nutrients 2021, 13. 10.3390/nu13092972 PMC8470649 34578848

[9] Souza S, Pereira AP, Prandini NR, Resende A, de Freitas EAM, Trigueiro TH (2022). Breastfeeding in times of covid-19: a scoping review. Revista Da Escola De Enfermagem Da U S P.

[10] Liu X, Chen H, An M, Yang W, Wen Y, Cai Z (2022). Recommendations for breastfeeding during coronavirus Disease 2019 (covid-19) pandemic. Int Breastfeed J.

[11] World Health Organization WHO. : *Breastfeeding and covid-19: Scientific brief, 2020*

[12] World Health Organization WHO. Indicators for assessing infant and young child feeding practices: part 2: measurement. World Health Organization; 2010.

[13] Binns CW, Fraser ML, Lee AH, Scott J (2009). Defining exclusive breastfeeding in Australia. J Paediatr Child Health.

[14] Greiner T (2014). Exclusive breastfeeding: measurement and indicators. Int Breastfeed J.

[15] Ladomenou F, Moschandreas J, Kafatos A, Tselentis Y, Galanakis E (2010). Protective effect of exclusive breastfeeding against Infections during infancy: a prospective study. Arch Dis Child.

[16] Dennis C-L, Brown HK, Brennenstuhl S (2017). The postpartum partner support scale: development, psychometric assessment, and predictive validity in a Canadian prospective cohort. Midwifery.

[17] Eslahi Z, Alimoradi Z, Bahrami N, Lin CY, Griffiths MD, Pakpour AH (2021). Psychometric properties of postpartum partner support scale—persian version. Nurs Open.

[18] Bruwer B, Emsley R, Kidd M, Lochner C, Seedat S (2008). Psychometric properties of the multidimensional scale of perceived social support in youth. Compr Psychiatr.

[19] Salimi A, Joukar B, Nikpour R (2009). Internet and communication: perceived social support and loneliness as antecedent variables. Q J Psychol Stud.

[20] Ingram J, Johnson D, Copeland M, Churchill C, Taylor H (2015). The development of a new breast feeding assessment tool and the relationship with breast feeding self-efficacy. Midwifery.

[21] Dennis C-L, Faux S (1999). Development and psychometric testing of the breastfeeding self-efficacy scale. Res Nurs Health.

[22] Marzieh Araban FF, Mehrjardi P, Shahry A, Montazeri (2016). The persian version of breastfeeding self-efficacy scale-short form (bses-sf): translation and psychometric assessment. Payesh (Health Monitor) Journal.

[23] Bertino E, Moro GE, De Renzi G, Viberti G, Cavallo R, Coscia A (2020). Detection of sars-cov-2 in milk from covid-19 positive mothers and follow-up of their infants. Front Pead.

[24] Hui LL, Yeung KH, Chow KM, Poon LC, Ip PL, Nelson EAS (2023). Breastfeeding challenges and opportunities during covid-19 in Hong Kong. J Paediatr Child Health.

[25] Shaker MS, Oppenheimer J, Grayson M, Stukus D, Hartog N, Hsieh EWY (2020). Covid-19: pandemic contingency planning for the allergy and immunology clinic. J Allergy Clin Immunology: Pract.

[26] Oncel MY, Akın IM, Kanburoglu MK, Tayman C, Coskun S, Narter F (2021). A multicenter study on epidemiological and clinical characteristics of 125 newborns born to women infected with covid-19 by Turkish neonatal society. Eur J Pediatrics.

[27] Ceulemans M, Foulon V, Ngo E, Panchaud A, Winterfeld U, Pomar L (2021). Mental health status of pregnant and breastfeeding women during the covid-19 pandemic—a multinational cross-sectional study. Acta Obstet Gynecol Scand.

[28] Ceulemans M, Hompes T, Foulon V (2020). Mental health status of pregnant and breastfeeding women during the covid-19 pandemic: a call for action. Int J Gynecol Obstet.

[29] Assarian F, Moravveji A, Ghaffarian H, Eslamian R, Atoof F (2014). The association of postpartum maternal mental health with breastfeeding status of mothers: a case-control study. Iran Red Crescent Med J.

[30] Lebel C, MacKinnon A, Bagshawe M, Tomfohr-Madsen L, Giesbrecht G (2020). Elevated depression and anxiety symptoms among pregnant individuals during the covid-19 pandemic. J Affect Disord.

[31] Scott JA, Binns CW, Oddy WH, Graham KI (2006). Predictors of breastfeeding duration: evidence from a cohort study. Pediatrics.

[32] Piankusol C, Sirikul W, Ongprasert K, Siviroj P (2021). Factors affecting breastfeeding practices under lockdown during the covid-19 pandemic in Thailand: a cross-sectional survey. Int J Environ Res Public Health.

[33] Pacheco F, Sobral M, Guiomar R, de la Torre-Luque A, Caparros-Gonzalez RA, Ganho-Ávila A (2021). Breastfeeding during covid-19: a narrative review of the psychological impact on mothers. Behav Sci.

